# Muscle Hypertrophy in a Newly Developed Resistance Exercise Model for Rats

**DOI:** 10.3389/fphys.2022.851789

**Published:** 2022-05-13

**Authors:** Hameed Al-Sarraf, Abdeslam Mouihate

**Affiliations:** Department of Physiology, Faculty of Medicine, Kuwait University, Safat, Kuwait

**Keywords:** strength training, fast-twitch muscle fiber, tunnel-pulley system, muscle hypertrophy, treadmill exercise

## Abstract

Clinical evidence suggests that resistance exercise exerts health benefit. The mechanisms underlying such health benefits is largely explored in experimental animals. Available experimental models have several shortcomings such as the need for noxious stimuli that could affect the physiological readouts. In this study, we describe a simple-to-use experimental model of resistance exercise. In this resistance exercise, rats pull pre-determined weights using a tunnel and pulley system. We show that resistance-exercised rats developed a larger pulling strength when compared to those seen in either control rats or in rats subjected to traditional treadmill exercise. Histological examination revealed that resistance exercise led to a larger fiber cross-sectional area in the plantaris muscle, but not in the gastrocnemius or the soleus muscles. Similarly, the percentage of type-II muscle fibers in the plantaris was increased in resistance exercised rats when compared to those seen in plantaris muscles of either control or treadmill-exercised rat groups. Furthermore, this resistance exercise led to a significant increase in the expression levels of the phosphorylated protein kinase B; a marker of muscle hypertrophy in the plantaris muscle. Such effects were not seen in treadmill-trained rats. In conclusion, we developed an experimental model that can be amenable for experimental exploration of the mechanisms underlying the beneficial effects of resistance exercise. We further provide evidence that this resistance exercise model enhanced muscle strength and muscle hypertrophy.

## Introduction

It is well known that both aerobic and resistance exercise have beneficial effects on human health (Bauman, 2004; Penedo and Dahn, 2005; Reiner et al., 2013). However, the metabolic changes that occur at cellular, tissue and organ levels vary considerably between these types of training (Atherton et al., 2005; Coffey et al., 2006; Coffey and Hawley, 2007; Egan and Zierath, 2013). Although both aerobic and resistance exercise can have beneficial effects on human health, their impact on the body vary based on the parameter of interest. For example, aerobic training more effectively reduces risk factors for cardiovascular diseases, whereas resistance training is more effective in improving basal metabolic rate while increasing muscle mass, and physical function (Egan and Zierath, 2013). Recently, the importance of resistance exercise has received greater attention. The majority of studies on resistance training are performed on human subjects. Working with human subjects is limited by such factors as poor control on nutrition and environment of subjects, ethical issues and difficulty in sampling and collection of tissues (Lowe and Alway, 2002; Kapp, 2006). Alternatively, using animal models of resistance exercise offers several advantages which include maintaining the animals under standard laboratory conditions, availability of several disease models for mechanistic exploration of the beneficial effects of resistance exercise, and ease of intrusive tissue sampling amenable to cellular and molecular explorations. Thus, developing an animal model of resistance exercise that closely resembles resistance training in humans is of great importance. During the past few decades, several experimental models of resistance exercise in rodents were developed (Lowe and Alway, 2002; Cholewa et al., 2014; Strickland and Smith, 2016). However, this has proven to be challenging since rodents are not normally inclined to lift weight unless stimuli with either negative or positive reinforcements were applied. Researchers have designed special methods to study resistance exercise in animals, such as surgical ablation (Goldberg, 1968), electrically stimulated squat (Tamaki et al., 1992), electrical stimulations on unconscious rats (Warren et al., 1998; Wong and Booth, 1988; Baar and Esser, 1999), passive stretch (Alway et al., 1989), operant conditioned squat (Wirth et al., 2003; Nicastro et al., 2012), planar extension in order to obtain food (Klitgaard, 1988; Łochyński et al., 2016) and ladder climbing (Yarasheski et al., 1990; Lee et al., 2004). Currently, the most widely used animal model of resistance exercise is the ladder climbing in rats. In this model, variable weights are attached to the tail of the rat which is trained to climb a ladder. This method appears to be the most reliable model for resistance exercise. However, it requires either food deprivation or electric shock to induce the rat to climb the ladder especially when heavy loads are applied (Yarasheski et al., 1990; Hornberger and Farrar, 2004; Silveira et al., 2011). In the present work, we describe a new experimental model of resistance exercise in which rats pull weights while walking through a tunnel. Pulling weight in a horizontal plane appears to be more natural to the animal, hence no electric shock was required even when the animals were pulling heavier loads. We assessed the validity of this model by comparing the strength and muscle hypertrophy of resistance exercise trained rats to the control and rats who ran on a treadmill. Here, we provide the first description of a model of resistance exercise which does not require positive or negative re-enforcements.

## Materials and Methods

### Animals

Adult male Sprague Dawley rats (∼10 weeks old) were obtained from the Animal Resources Center (ARC) at Kuwait University (KU). The rats were kept at an ambient temperature of ∼23°C under 12-h light-dark cycle (lights on from 7:00 a.m. to 7:00 p.m.) with free access to pelleted chow and water. Rats were randomly divided into three groups (6 animals each); a control group (walking the track without weights) a resistance exercise group and a treadmill exercised group. Ethical approval was obtained from the Health Sciences Center Ethics committee of KU, and the rats were treated in accordance with guidelines on the humane handling of experimental animals. The resistance exercise protocols were performed during the animals’ dark cycle.

### Resistance Exercise Apparatus

**FIGURE 1:**
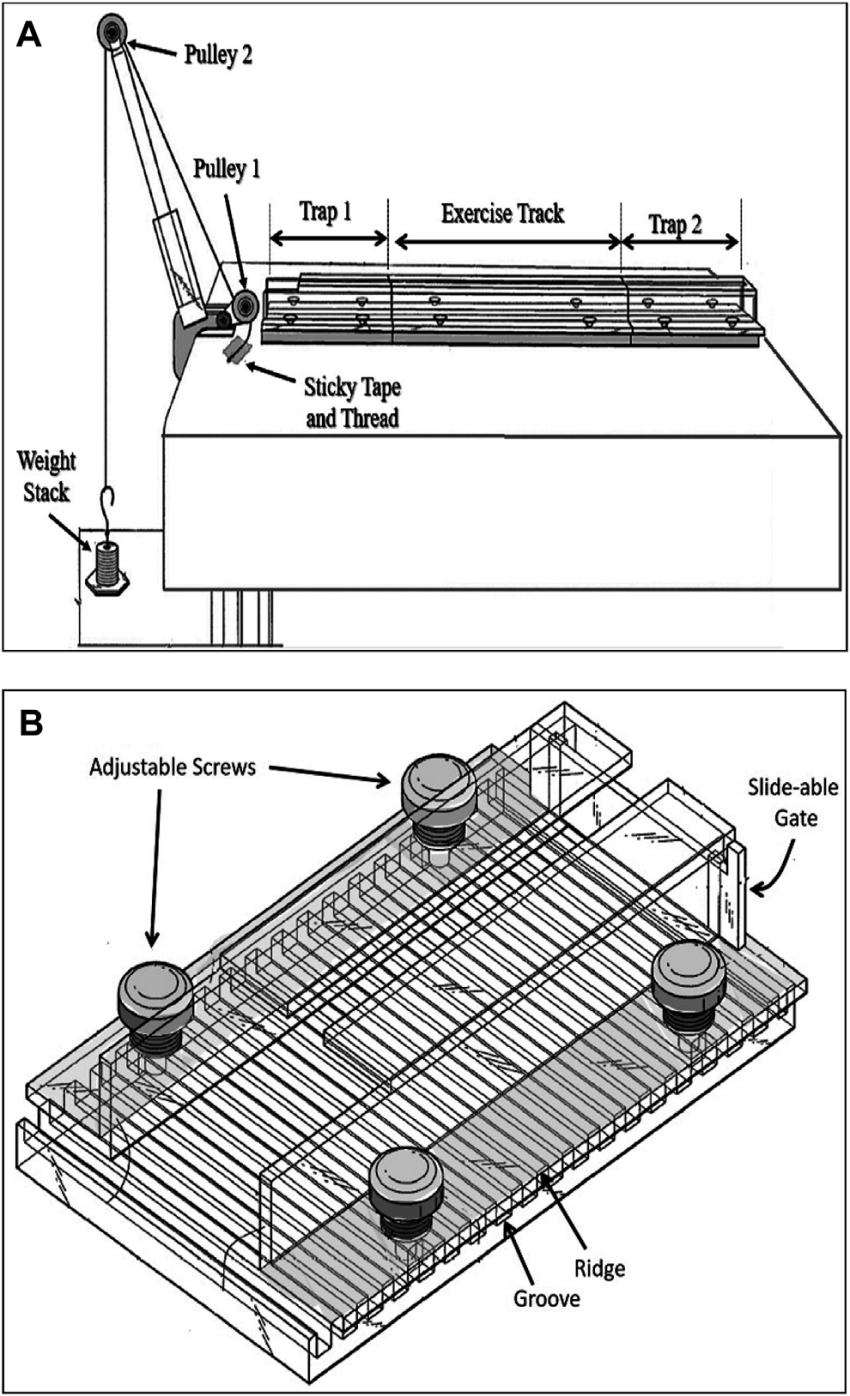
The rat resistance exercise system. **(A)** The three sections-transparent tunnel positioned in front of a two pulley tower. Once the animal enters the trap 1 compartment **(B)**, a strong cotton rope that runs over two pulleys is tied to a weight stack from one end and secured to the tail of a rat at the other end. The trap compartment is adjustable for width (4.5–8 cm) and height (4–7 cm) through movable sidewalls and placing of various thickness washers, respectively. It has a slide-able gate in front.

The resistance exercise apparatus was designed in our laboratory and built in the mechanical workshop of the Faculty of Medicine, Kuwait University (Patent No.: US 10,426132 B2). This apparatus consists of three transparent Plexiglas sections forming a tunnel that has two identical sections (trap 1 and trap 2) which are placed at both ends of a longer middle section (exercise track). Altogether, these three sections form a continuous tunnel. This tunnel is placed on a table in front of a two-pulley tower (Figure 1A). The track is the main section in which the animal pulls a pre-determined weight attached to its tail. This section can be of three different sizes based on the type of resistance exercise required. The length of the track section can be either 20 cm that allows the rat to complete the run by taking 4 or 5 steps, targeting muscle strength, 80 cm allowing 9 to 12 steps targeting muscle hypertrophy, or 120 cm allowing 15 to 22 steps which is designed to improve muscle endurance. The two identical trap sections (Figure 1B) function as traps for the animal, each one is 25 cm long, and they are interchangeable with each other after each weight pulling run. Therefore, the entire system when in use (trap 1 + exercise track + trap 2) line up to form either a 70 cm, or a 130 cm, or a 170 cm long tunnel. The tunnel can be tilted at different angels of inclination which enables the researcher to apply the pulling load at different degrees, recruiting the working muscles at various angles. In this study, we used the 130 cm long system at 0-degree inclination. The width of each section is adjusted by adjustable screws (Figure 1B) allowing the sidewalls of the tunnel to move apart for a wider tunnel or come close to each other to form a narrower tunnel. The height of each tunnel section can also be adjusted by placing washers of various thicknesses at the adjustable screws. These adjustments allow the tunnel to accommodate rats of various sizes (150–750 g). Throughout the study, the rats grew considerably, their changing body size was accommodated in the system by the above adjustments for body weights as small as 350 g at the beginning of study up to 630 g body weight on the day of sacrifice. The floor of all three sections of the tunnel has 0.8 mm deep grooves that are separated by 0.7 mm ridges of 0.5 mm width. This allows optimal grip of the animal when lifting heavy weights (Figure 1B). The three sections lined up on a table that has a height of 120 cm. A two-pulley tower secured to the table at one end to allow using pulling line greater than table height (Figure 1A). At the front end of each trap section, there is a sliding gate to prevent the animal from moving out of the section (Figure 1B).

### Resistance Training Procedure

**FIGURE 2:**
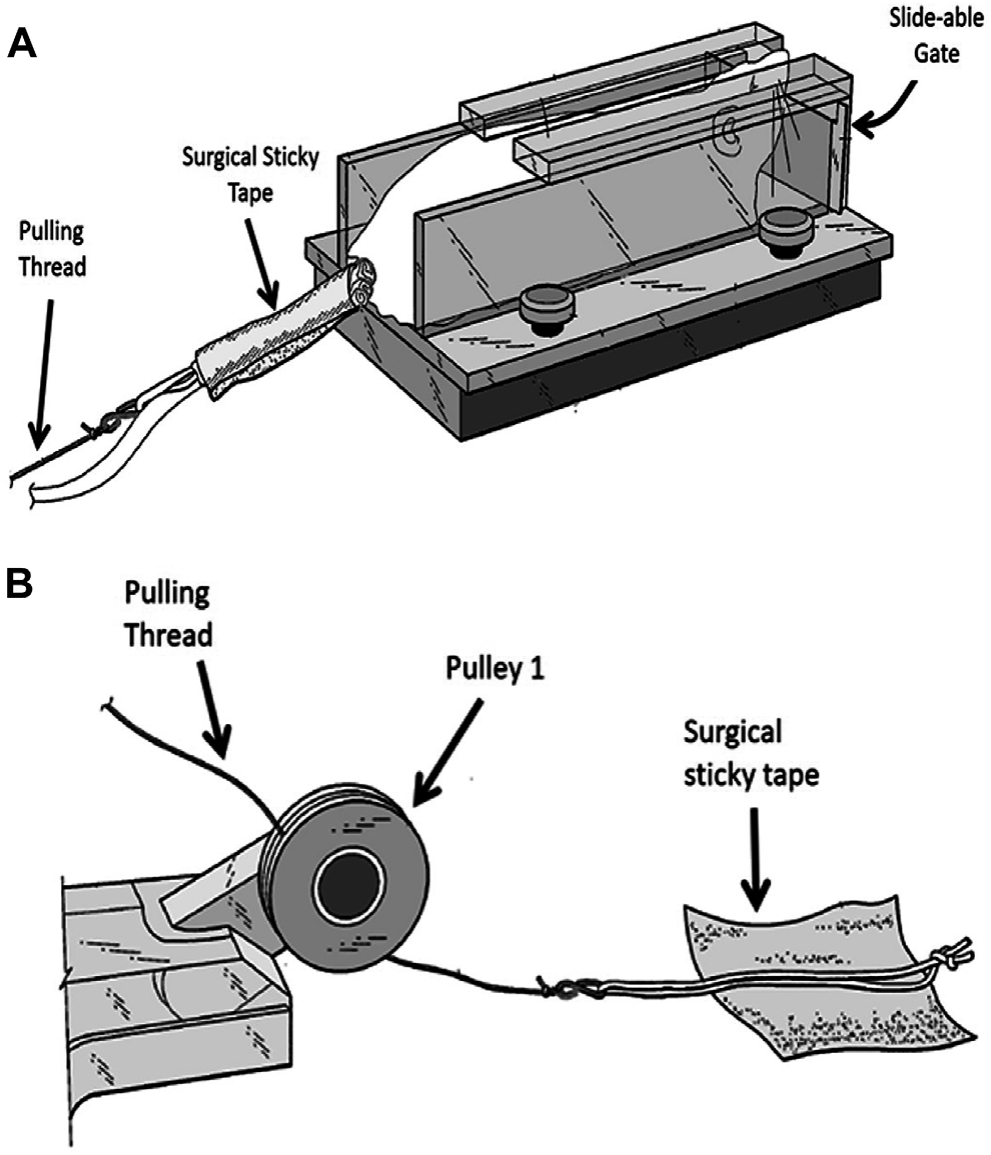
Securing the animal to the weight stack. **(A)** A rope is secured to rat’s tail by wrapping the sticky tape around the tail. **(B)** A soft cotton rope placed in the middle of a square shaped sticky hypo-allergic tape. Two edges of the tape are folded to allow easy removal of the sticky tape at the end of the experiment.

At the start of each resistant training, the rat is placed near the entrance of the trap 1 section. Once the rat enters this section it will be trapped by a closed sliding gate in the front of this section (Figure 2A). The rear end of the rat is exposed allowing the researcher to hold the rat in place with his/her hand while securing the thread on its tail. A long thread which runs under pulley 1 and over pulley 2, attached to an adjustable weight stack resting on the floor (Figure 1A) is secured to the tail of the animal using a hypoallergic sticky tape (Figure 2). It takes approximately 7 s to secure the rope on rat’s tail with the sticky tape. This part of the experiment is important for minimizing the stress to the animal. The surgical sticky tape allows the animal to pull heavy weights with no apparent pain or injury. The weight stack provides pulling resistance range between 50 and 1750 g at increments of 50 and 100 g.

At the start of the exercise, the gate is opened and the animal moves through the tunnel pulling a pre-determined weight attached to its tail. When it reaches the end section (trap 2), this section containing the animal is moved to the beginning of the tunnel by exchanging positions with trap 1, hence placing the rat at the beginning of the three-section tunnel to start a new cycle. Throughout the experiment, the animal was handled only when picked up from the cage and for a very short duration when securing/removing the sticky tape to/from the tail.

### Resistance Training Protocol

Rats were trained four times per week (Sundays, Mondays, Wednesdays, and Thursdays) for 11 weeks. Each training session started with four warm-up runs with a weight equivalent to 25% body weight followed by a progressive overload at 50%, 75% in the first week and then in subsequent weeks the progressive overload was increased up to 150% of the body weight. Each animal performed 14 to 16 runs with resting period of 20–60 s between runs. The resting period was carefully chosen depending on the amount of weight pulled by the animal as greater weights required more rest. Negative reinforcement such as electric shock or strong poking were not required at any time during the experimental procedure. Occasionally, rats were encouraged by sound or soft touch on their back. Gradually, these signals became cues for the animal to move through the tunnel as a behavioral adaptation.

### Treadmill Protocol

Rats trained four days per week on a treadmill (Columbus Instruments, Exer-3R treadmill, Columbus, OH, United States) for 11 weeks. The running speed and time were progressively increased up to 25 m/min for 30 min.

### Testing of Pulling Strength

On the first day of the study, all animals were tested for their pulling strength in the system. An elastic band was calibrated using a digital scale. To test the strength, the calibrated elastic band was secured at one end at a fixed position. The other end of the band was secured to the tail of the rat. The distance that the rat stretched was inferred to the equivalent weight in grams. Each animal pulled the elastic band through the tunnel three time and the average distance was established for that animal. All animals were tested for their strengths once every two weeks.

### Tissue Collection

Rats were weighed once weekly throughout the duration of this study. After 11 weeks, rats were deeply anesthetized with 1.5 mg/kg of urethane [(i.p.), Sigma Chemical Co., St. Louis, MO, United States] and transcardially perfused with ice-cold phosphate buffered saline solution (in mM; NaCl 137, Na_2_HPO_4_ 10, KCl 2.7, KH_2_PO_4_ 1.8). Samples of muscles of the hind limb (*soleus, plantaris and gastrocnemius*) which are most involved during this resistance exercise were harvested, weighed on a microbalance (Mettler Toledo MT5) then were either snap frozen in liquid nitrogen and stored in −80°C freezer for western blot analysis, or post-fixed in neutral-buffered formalin solution for immuno-histochemical exploration.

### Immunohistochemistry

To explore muscle hypertrophy, muscle fiber diameters were measured. The collected muscles, soleus, plantaris and the medial part of the gastrocnemius were cut at mid-belly, fixed in 10% neutral buffered formalin, embedded in paraffin blocks cut at 5 µm thickness and mounted on saline-coated slides as previously described (Kalakh and Mouihate, 2019). Briefly, muscle sections were incubated with a primary mouse monoclonal antibody anti-myosin type II (1:1000, Sigma-Aldrich) followed by a secondary polyclonal antibody anti-mouse IgG tagged with Alexa fluor 488 (1:400; Thermofisher). Four sections from each muscle were randomly sampled. Images of the cross sections of sampled muscles were acquired using a confocal microscope (Zeiss LSM 700 META microscope). Eight fields at magnification of 20x were randomly selected and images were acquired and analyzed using ImageJ (Schneider et al., 2012). For each muscle, 250–300 muscle fiber cross sectional areas were measured. For muscle typing, all muscles fibers (immunostained for the fast fibers or unstained) in aquired images were counted and the percentage of fast fibers (Myosin type-II immunoreactive fibers) was calculated.

### Western Blot

Muscle samples were homogenized in a lysis buffer (MOPS, 20 mM; KCl, 150 mM; Mg Acetate, 4.5 mM; Triton X, 1%) containing protease inhibitor cocktail tablet (Roche Applied Science, Mannheim, Germany) and centrifuged at 12,000 g for 15 min at 4°C. The supernatant containing proteins was then collected and protein levels were assayed using bicinchoninic acid protein assay (Pierce Chemical Co., Rockford, IL, United States). These proteins were separated using SDS-PAGE as described previously (Kalakh and Mouihate, 2017). Briefly, proteins (60 µg/well) extracted from either gastrocnemus, soleus or plantaris were separated on polyacrylamide gel and electroblotted onto a nitrocellulose membrane. The membrane was then washed and exposed to a primary rabbit polyclonal antibody anti-phosphorylated AKT (Santa Cruz Biotechnology, Santa Cruz, CA, United States; 1:500). After washing the primary antibody, the membrane was exposed to a horseradish peroxidase-tagged secondary anti-rabbit polyclonal antibody (Santa Cruz Biotechnology, Santa Cruz, CA, United States; 1:1000). The nitrocellulose membrane was exposed to an enhanced chemiluminescence solution (ECL prime GE Healthcare Life sciences, United States) and revealed using a Kodak X-Omat film. The membranes were subsequently incubated with mercaptoethanol-containing buffer solution to remove the antibodies, washed and re-exposed to a primary mouse monclonal antibody anti-AKT (total AKT: tAKT) (Cell Signaling Technology, Danvers MA, United States; 1:2000). The membranes were then washed and exposed to a horseradish peroxidase-tagged secondary anti-mouse (Invitrogen/Thermofisher, United States; 1:2000) and exposed to ECL as desctribed earlier. The optical density was determined by measuring the area under the curve of immunoreactive bands and used a semi-quantitative measure of protein levels. Expression of phosphorylated protein kinase B (Akt) was used as a marker of muscle hypertrophy (Grzelkowska et al., 1999; Anthony et al., 2000). The areas under the curve of the optical density (OD) profiles of pAKT- and tAKT-immunoreactive bands were determined using image J. The ratio of the OD values of pAKT/tAKT were calculated and presented as a fold change of their corresponding values obtained in control animals.

### Statistical Analysis

Western blot data were compared using unpaired student’s *t*-test. Data related to body weight gain and pulling strength were compared using repeated mesure ANOVA while those related to muscle hypertrohpy (immunoflurescence) were compared using one way ANOVA followed by a Student-Newman Keuls *post-hoc* test. Statistical significance is declared when the “*p”* value was less than 0.05.

## Results

### Body Weight Gain

**FIGURE 3:**
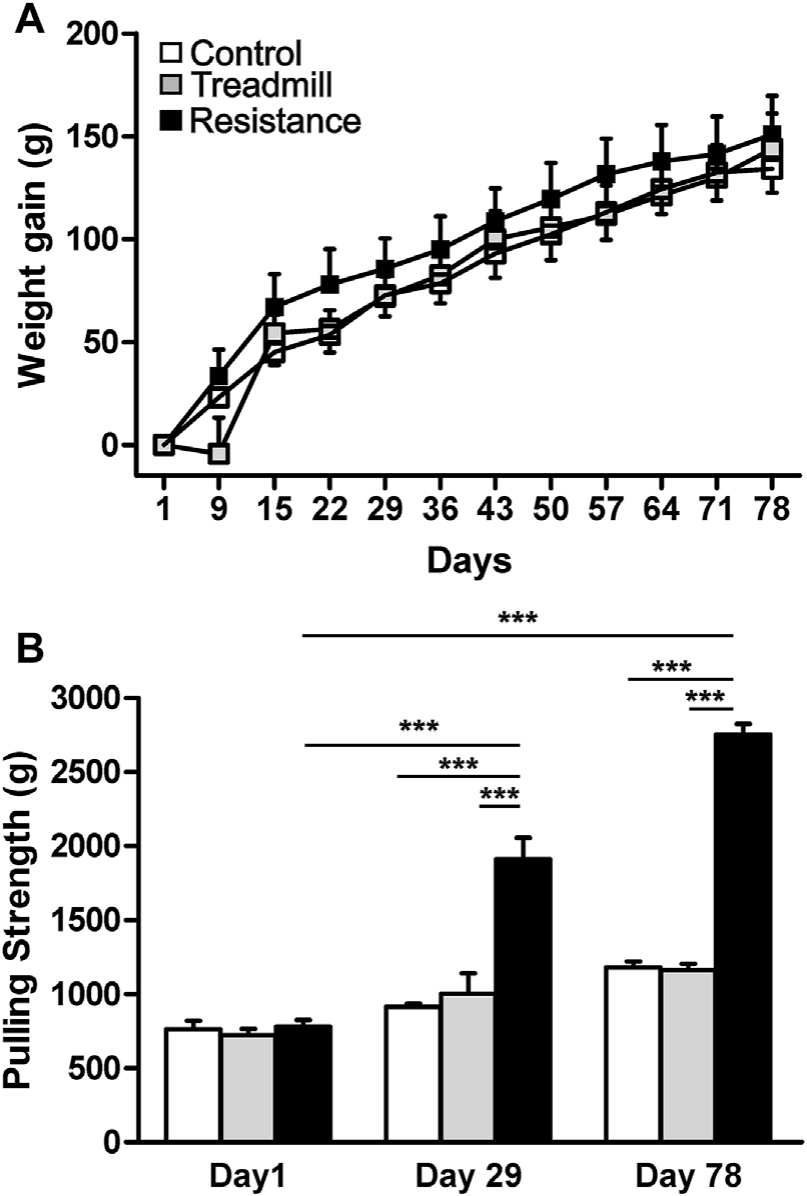
Effect of exercise on body weight gain and pulling strength. Upper panel **(A)** shows the weight gain in control rats (open) and rats subjected to either treadmill exercise (gray) or resistance exercise (black). There was no significant difference in the body weight gain between control rat group and those of resistance exercised and treadmill-exercised rats (*p* >0.05). The graph bar in the lower panel **(B)** illustrates the pulling strengths in control rats and rats subjected to either resistance exercise or treadmill exercise. Resistance exercise group showed greater strength when compared to either the control rats or the rats running on the treadmill (***p <0.001). There was significant increase in strength of resistance exercised rats from day1 to day 29 (***p <0.001) and day 78 (***p <0.001).

At the start of the study the animals of similar age (∼10 weeks old) were randomly assigned to groups. The average body weights were not significantly different between the groups; control = 436.3 ± 10.5 (g), treadmill (TM) = 407.0 ± 13.3 (g) and resistance exercise (RE) = 430.7 ± 11.0 (g), (*p* > 0.05). On the day of sacrifice (∼21 weeks old) the body weighs were; 570.5 ± 18.4 (g) for control, 550.8 ± 23.9 (g) for TM and 581.5 ± 14.2 (g) for RE, respectively. The resistance exercise and treadmill training had no significant effect on body weight gain of the animals when compared to the control (*p* >0.05), (Figure 3A).

### Test of Pulling Strength

The average pulling strength of three attempts for each animal using an elastic band was established at three time points during the course of the study (Figure 3B). At the start of the study, day1, there was no significant differences in the pulling strength between the three rat groups (*p* >0.05). Compared to the day1, the animals in the RE group showed significant increases in pulling strength by 152 and 263% at days 29 and 78 respectively (*p* <0.001). At day 78, the pulling strength of RE group was higher than that of the control and TM rat groups by 135 and 141% respectively (*p* <0.001).

### Muscle Weights

**FIGURE 4:**
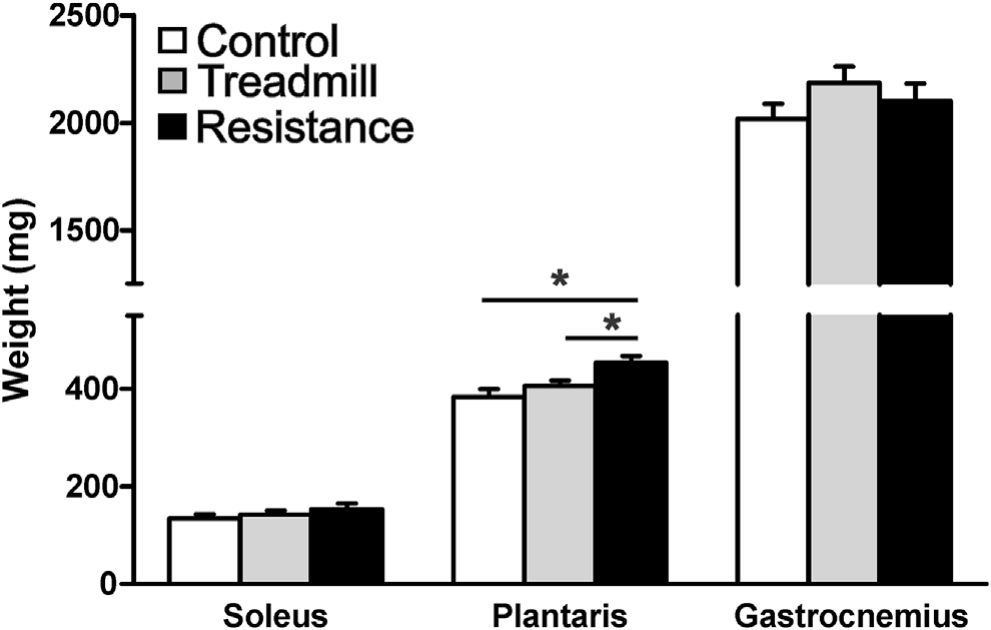
Resistance exercise increases the mass of the plantaris muscle. The graph shows wet weight of the hind limb muscles; soleus, plantaris and gastrocnemius in rats walking the track without pulling a load (control), running on treadmill, and rats subjected to 11 weeks of resistance exercise. The resistance exercise significantly increased the mass of the plantaris muscle when compared to those of control and treadmill (*p <0.05), while it had no significant effects on masses of either soleus or gastrocnemius (*p* >0.05).

Figure 4 shows the wet weights for the hind limb muscles in the resistance exercise group compared to their corresponding muscles in control and treadmill groups. Resistance exercise had no significant effect on the masses of either soleus and gastrocnemius muscles (*p* >0.05). Whereas, resistance exercise led to a significant increase in the mass of the plantaris (*p* <0.05).

### Fiber Cross Sectional Area (FCSA) and Fiber Types

**FIGURE 5:**
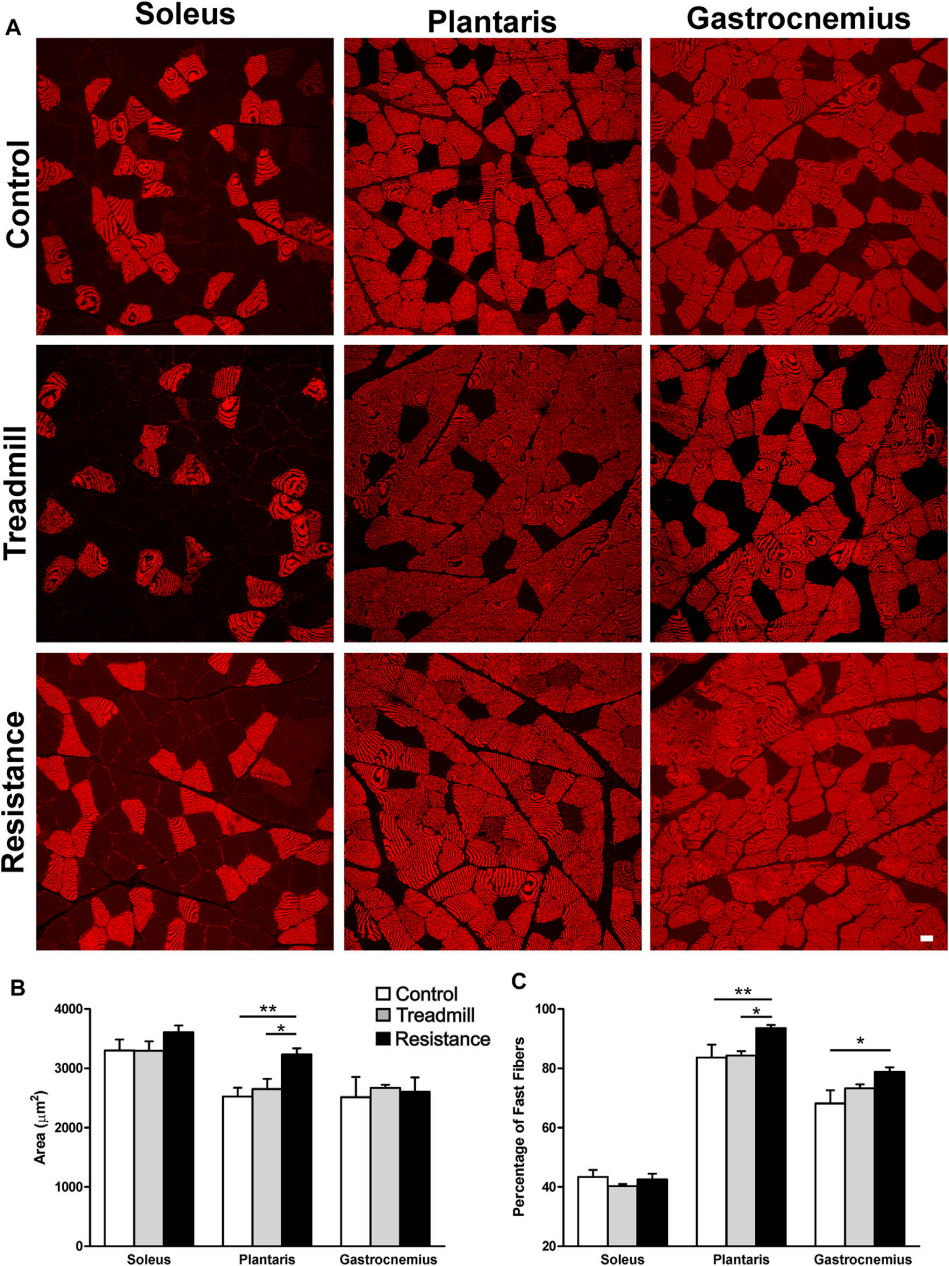
Impact of resistance exercise on the muscle fiber cross sectional areas (FCSA) and percentage of fast muscle fibers. **(A)** Micrographs illustrate immunofluorescent detection of fast muscle fibers in soleus, plantaris and gastrocnemius muscles of control and exercised rats. **(B)** The graph bars show FCSA analysis of muscle fibers in the soleus, plantaris and gastrocnemius in the control rats (open bars) or rats subjected to 11 weeks treadmill exercise (shaded bars) or 1 weeks of resistance exercise (solid bars). Resistance exercise led to a significant increase in the FCSA in the muscle fibers of the plantaris but not in those of the gastrocnemius or the soleus. **(C)** The graph bars show that resistance exercise led to a significant increase in the percentage of muscle fibers in both the plantaris and the gastrocnemius muscles. This effect was not seen in the soleus muscles. Data are presented as mean ± SEM. *p <0.05, **p <0.01.

Figures 5A,B shows the FCSA of sampled muscles. Eleven weeks of resistance exercise led to a larger FCSA in the plantaris (3234 ± 102 μm^2^)*,* compared to the TM (2650 ± 171 μm^2^; *p* <0.05) and the control rat group (2524 ± 149; (*p* <0.01). There was no significant effect of resistance exercise on FCSA of either gastrocnemius or soleus muscles. Immunostaining for the fast (type II) muscle fibers revealed that the plantaris muscle has the greatest percentage of fast fibers when compared to the gastrocnemius and soleus muscles (Control: plantaris 84%, gastrocnemius 68% and soleus 43%; Figure 5C). Resistance exercise led to a significantly greater percentage of fast twitch muscle fibers in the plantaris when compared to control and treadmill-exercised rat groups. Resistance exercise induced a slight but statistically significant increase in the percentage of fast twitch muscle fibers in the gastrocnemius muscle when compared to the control rat groups. On the other hand, resistance exercise had no significant effects on the percentage of fast twitch fibers in the soleus.

### Impact of Resistance Exercise on Markers of Hypertrophy in Hind Limb Muscles

**FIGURE 6:**
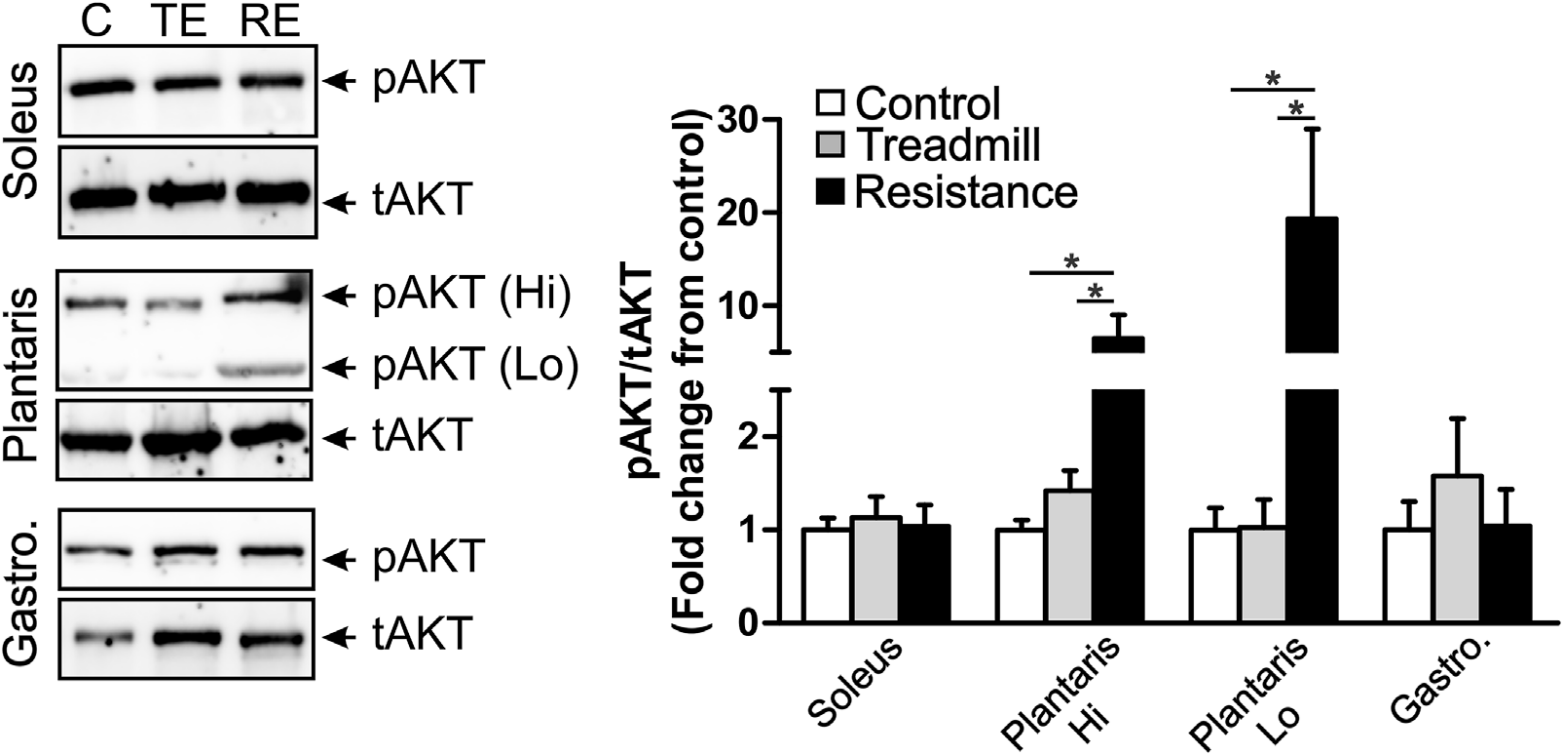
Resistance exercise enhances the expression levels of the phosphorylated fraction of AKT in the plantaris muscle. Left panel shows immunoblots of phosphorylated AKT (pAKT) and total AKT (tAKT) detected in the soleus (top micrograph), the plantaris (middle micrograph) and the gastrocnemius (bottom micrograph). There were two IR bands with a high (90 KD) and low (64 KD) apparent molecular weights in the plantaris muscle. One pAKT-IR band was seen in the soleus muscle at the apparent molecular weight of 64 KD and one pAKT-IR band was seen in the gastrocnemius at the apparent molecular weight of 66 KD. One major tAKT IR band was detected in all three muscles. The right panel shows bar graph illustrating semi-quantitative analysis of the expression of the phosphorylated fraction of AKT relative to the total AKT (pAKT/tAKT) in the soleus, plantaris (high MW: Hi and low MW: Lo) and gastrocnemius muscles of rats under control condition (C; *n* = 5) or subjected to either treadmill (TE; *n* = 5) or resistance exercises (RE; *n* = 5). Resistance exercise led to a significant increase in the expression of the fraction of pAKT in the plantaris, but not in the gastrocnemius or the soleus muscles. Data are presented as mean ± SEM. *p <0.05.

We explored the effect of exercise on the expression of levels of the phosphorylated form of AKT (pAKT), an indicator of muscle hypertrophy (Hornberger and Farrar, 2004). We observed two immunoreactive (IR) bands with a high (90 KD) and low (64 KD) apparent molecular weights in the plantaris muscle (middle micrograph in Figure 6), while we observed only one IR band (66 KD) in the gastrocnemius (bottom micrograph in Figure 6) and the soleus (64 KD; top micrograph in Figure 6). The total AKT (tAKT) was not significantly affected by either treadmill or resistance exercise in all muscles (data not shown). Resistance, but not treadmill exercise, led to a significant increase in the expression levels of the phosphorylated fraction of both 90 KD and 64 KD IR band in the plantaris. Such effect of exercise was not seen in either the soleus or the gastrocnemius. It appears that the muscle fibers in the plantaris were highly sensitive to the resistance exercise.

## Discussion

To our knowledge, this is the first detailed description of an experimental resistance exercise using pulleys and tunnel system for weight pulling in rodents. To validate this experimental model, we tested the pulling strength as well as explored skeletal muscle hypertrophy. The resistance exercise resulted in the increase in pulling strength and induced muscle hypertrophy. To examine muscle hypertrophy, we selected three ankle extensor muscles as ankle extension seems to be actively involved in the walking movement of the rats undergoing pulling against weights through the tunnel as well as running on the treadmill. We showed a progressive improvement in pulling strength and observed muscle fiber hypertrophy in the plantaris muscle which was revealed by a larger muscle mass and a larger fiber CSA in the resistance exercised group when compared to that of control and treadmill groups. In addition, the change in muscle fiber composition observed in both plantaris and gastrocnemius may also contribute to the increased in pulling strength in our model. We also provide cellular and molecular proof that this experimental resistance exercise model resulted in increased expression of an intracellular hypertrophic marker, also mostly in the plantaris muscle. These effects were not observed in rats that ran on treadmill and those which walked the tunnel without resistance.

Our model induced ∼11% increase in muscle mass of plantaris while it had no significant effect on masses of either the gastrocnemius or the soleus. The ladder climb model increased the mass of flexor hallucis longus muscle by ∼17% (Lee and Farrar, 2003) and ∼23% (Lee et al., 2004) but did not affect other planar flexor muscles groups (soleus, plantaris and gastrocnemius). The selective increase in the plantaris mass in our model was associated with a corresponding increase in the muscle fiber CSA of about 26%. Similar to our findings, resistance training in SD rats on a loaded running wheels showed increased in plantaris fiber CSA by ∼20% and increase in its mass by ∼17% (Ishihara et al., 1998). The muscle fiber hypertrophy observed in our study is comparable to muscle fiber hypertrophy seen in more invasive methods used in previous studies for various durations. Indeed, electrical stimulation-induced squat model of resistance exercise in rats for 8 weeks (Ho et al., 1980), 15 weeks (Tamaki et al., 1992) or 36 weeks (Klitgaard, 1988) led to an increase in the size of muscle fibers by ∼12%, ∼20 and ∼30% respectively. Planar extension squat to access food against a load resulted in 24% increase the muscle mass (Klitgaard, 1988) and 23% increase in fiber CSA (Zanchi et al., 2010). Using the same weight lifting training model Lochynski *et al.*, found improvement in tetanic contraction produced by the gastrocnemius while no change in muscle mass was detected (Łochyński et al., 2016). Using the ladder climb model, Hornberger and Farrar (Hornberger and Farrar, 2004) reported 23% increase in flexor halluces longus muscle mass after 8 weeks of exercise. Most animal resistance training models generally train between 6–12 weeks. However, using the ladder climbing model for a longer duration (26 weeks), Duncan and colleagues (Duncan et al., 1998) reported 36% muscle fiber hypertrophy in the plantaris muscle, 32% in soleus muscle and 26% in rectus femoris muscle. Our model and the training protocol was based on the overload principle and it was designed for the number of sets, repetitions and rest periods between sets to closely resembled that of a typical resistance training program for humans. However, we aimed at a greater frequency than human programs. In a typical resistance training programs in humans, a muscle group is trained once or twice per week (Duncan et al., 1998; Booth and Thomason, 1991), while in our model the training was for 15 min durations four times per week. It should be noted that rodents require greater amount of work than humans to achieve similar hypertrophic effects of the resistance exercise (Klitgaard, 1988). Therefore, a greater frequency of training maybe needed to induce muscle hypertrophy in rodents. It is rather surprising that after 11-weeks of training our model failed to induce muscle fiber hypertrophy in the gastrocnemius muscle which is shown to be sensitive to resistance training (Zoladz, 2019). In our model in order to pull against the load, the rat moves in the horizontal axis while gripping against the floor of the track. Perhaps this mechanical maneuver recruits the plantaris more than the gastrocnemius or the soleus muscles. The mechanical maneuver of our model closely resembles a recently established model of resistance exercise in which mice pull a loaded cart in horizontal plain (Zhu et al., 2021). This model also showed hypertrophy in the plantaris muscle but not in the gastrocnemius.

We also observed that resistance exercise affected muscle composition favoring increase in fast-twitch muscle fibers in the gastrocnemius and plantaris muscles. Compared to the soleus muscle, both gastrocnemius and plantaris have greater proportion of fast-twitch muscle fibers. Therefore, some of the increased strength observed in the resistance exercise group could be attributed not only to the hypertrophy of the plantaris, but also to the increase in fast-twitch muscle fibers after adaptation to the resistance exercise. In humans it has been shown that the increase in strength during the first two months of resistance exercise is mainly due to neuronal adaptation (Klitgaard, 1988). Similar adaptation has been suggested in rodents (Roy et al., 1997; Komi, 1986). The increase in the pulling strength displayed by the resistance exercised rats over the 11-week training program of the present study closely resembles the strength improvements in lifting ability shown by humans over the course of a resistance training program.

We used a pre-calibrated elastic band to assess the pulling strength. To avoid producing exercise effect in the control animals, we used an elastic band which allowed us to test the pulling strength with limited number of runs. In previous studies, in ladder climbing rats, the grip strength was found to increase by 13–20% (Kim et al., 2015; Aamann et al., 2019). Using the elastic band to assess the pulling strength, we observed ∼129% increase in pulling strength in the resistance exercise group from day1 to day 78, when corrected for the body weights. It must be noted that the test of strength utilized in our study evaluates the pulling strength of three runs only, whereas the resistance exercise group was adapted in pulling a progressively increasing weights for up to 16 runs. It is highly likely that if we had tested the full 16 run pulling ability of the groups that resistance exercise group would have performed even better. By using our method, the observed improvement in the pulling strength cannot establish the individual contribution of each of the three studied muscles. Future studies will address this issue by measuring tetanic developed tensions by individual flexor muscles. These explorations are beyond the scope of the present study, which was mainly designed to determine the effect of resistance exercise on overall muscle strength and muscle hypertrophy.

To further validate this experimental model of resistance exercise, we explored the impact of such exercise training on a marker of protein synthesis and growth, protein kinase B (Akt) (van Weeren et al., 1998). Skeletal muscle hypertrophy is initiated by a series of intracellular signaling pathways one of which leads to the phosphorylation of protein kinase B, Akt (for review see (Schiaffino and Mammucari, 2011) and (Yoon, 2017). In our study, we provide experimental evidence that resistance exercise but not treadmill exercise induced an increased phosphorylation level of Akt-1 in the plantaris muscle. We noticed that there were two pAKT IR bands in the plantaris, but not in the gastrocnemius or the soleus. The mechanism underlying the selective increase of two pAKT IR bands in the plantaris is unclear. This peculiar observation might be due to the heavy involvement of the plantaris in this model of resistance exercise. More studies are required to delineate the physiological significance of this selective phosphorylation of two AKT isoforms in the plantaris.

These data support the possibility that our newly developed experimental model of resistance exercise induces hypertrophy in the plantaris muscle. This possibility is comforted by the observation that this resistance exercise induced an increase in the size of the muscle fibers in the same muscle. It is peculiar that such intracellular changes and increased size of the muscle fibers did not occur in other, equally important hind limb muscles, namely the gastrocnemius and the soleus, nor did it occur when rats were subjected to running on a treadmill. Perhaps different muscles adopt different strategies to increase their pulling strengths. For example, we observed an increase in the proportion of fast-fibers in the gastrocnemius without a major change in cellular markers of hypertrophy. It is noteworthy that the main objective of this study was to provide a proof of principle for the hypertrophic effect of a newly developed resistance exercise model at the intracellular levels. However, the results of this study open questions on the cellular and molecular mechanisms underlying the differential adaptation of synergistic muscles during resistance exercise.

### Advantages and Limitations of Our Pulley System

There are several advantages to the resistance exercise model described in this study. It closely resembles resistance exercise in humans, allows full control on all aspects of the resistance exercise such as frequency, load, repetitions, resting time, and more importantly it does not require negative or positive reinforcements. Also, an important feature of the tunnel and pulley system is that it does not require a training period. When the rats are first introduced to the tunnel, they naturally move through it even when heavier weights were applied. In fact, the pulling stimulus on the tail of the rat seems to prompt an innate response to move forward. In comparison, the ladder climb model requires several training sessions (Yarasheski et al., 1990; Duncan et al., 1998; Hornberger and Farrar, 2004; Strickland and Smith, 2016). This is an important feature of the tunnel and pulley system which does not constrain the researcher to exclude animals that are difficult to train. Hence, when studying various aspects of resistance exercise, this exclusion could affect the outcome of the study. Also, acute effects of exercise can be studied at ease. Another advantage of the tunnel and pulley system is that the rat performs each run relatively fast. Each run (9–12 steps) took approximately 6–9 s to complete indicating a comparable tempo (repetitions/time) to the human resistance exercise. This is compared to approximately 39 s in untrained and 15 s in 3-days trained rats climbing a ladder reported by (Cassilhas et al., 2013).

In our model, there is no direct handling of the rat during the exercise sessions. In contrast, the weight pulling mouse model of (Zhu et al., 2021), and the ladder climb model require repeated handling of the animals at the end of each run and placing them at the starting point. This repeated handling likely causes an additional stress. Furthermore, the experimenter occasionally delivers electric shocks to induce animal’s run. By comparison, no electric shock was needed in our experimental model. Perhaps for these reasons, the rats showed no overt sign of stress (i.e. no droppings, no vocalization, innate tendency to move into the tube). They do not need extra noxious stimuli (electric chock for ex.) to induce their pulling efforts. However, in order to confirm this apparent lack of stress in our experimental model, further studies on stress markers are required.

Our experimental model has its own limitations. Indeed, there is a sustained tension generated by the weights that requires constant recruitment of the pulling muscles. This usually does not occur in human resistance exercise. In human, each repetition involves concentric and eccentric cycles interspersed with moments of less tension. This limitation could be resolved by addition of a one-way gear system at the pulleys so that muscle recruitment stop when the animal is in stationary position. Another limitation is the potential grip failure affecting the pulling force of the rat. However, even when we applied a bigger load, the rats could hold still in their positions without sliding backwards. This is an indication of sufficient grip for holding. But the grip may not be sufficient enough for pulling. Therefore, the question of what fails; is it the grip or the pulling muscles is still an open question.

## Conclusion

This is the first detailed description of an experimental model of resistance exercise in rats using tunnel and pulley. Currently, several experimental models for resistance exercise exist but since inducing animals to lift weight is a great challenge, these models use either negative or positive re-enforcements and often require frequent handling which is stressful to the animal. Here, we designed an experimental model using the innate tendency of rats to move through tunnels to pull weights without the need for positive or negative re-enforcements. We further give a proof of principle that this experimental model led to increase in strength and induced muscle hypertrophy in a key muscle required for weight pulling. This experimental model can be adapted to study the impact of resistance exercise on several physiological parameters of different body organs. Such experimental explorations overcome the limitations in human’s studies as it allows the experimenter to perform invasive procedures such as tissue and blood sampling.

## Data Availability

The original contributions presented in the study are included in the article, further inquiries can be directed to the corresponding author.
